# Antifungal effect of the effect of *Securigera securidaca* L. vaginal gel on *Candida species*

**DOI:** 10.18502/cmm.5.3.1744

**Published:** 2019-09

**Authors:** Atefeh Raesi Vanani, Masoud Mahdavinia, Heibatullah Kalantari, Saeed Khoshnood, Maryam Shirani

**Affiliations:** 1 Student Research Committee, Ahvaz Jundishapur University of Medical Sciences, Ahvaz, Iran; 2 Toxicology Research Center, Ahvaz Jundishapur University of Medical Sciences, Ahvaz, Iran; 3 Department of Pharmacology and Toxicology, School of Pharmacy, Ahvaz Jundishapur University of Medical Sciences, Ahvaz, Iran; 4 Student Research Committee, School of Medicine, Bam University of Medical Sciences, Bam, Iran

**Keywords:** Antifungal effect, Candida albicans, Candidiasis, Vaginal gel

## Abstract

**Background and Purpose:**

*Candida* species are opportunistic fungi, capable of causing acute and chronic infections in the gastrointestinal tract, vagina, and oral mucosa, among which *Candida albicans* is the most important species. The *Securigera securidaca L.* is used as an antiseptic to treat some diseases in traditional Iranian medicine. The aim of this study was to evaluate the antimicrobial activity of *S. securidaca* extracts and vaginal gel against different *Candida* species.

**Materials and Methods:**

Antifungal effects of different extracts and vaginal gel of *S. securidaca* were investigated against *Candida* species. By using well diffusion test, different concentrations of the collected *S. securidaca* extracts and vaginal gel were examined to test their antifungal activity against *C. albicans*, *C. parapsilosis*, and *C. krusei*.

**Results:**

The ethanol extract and vaginal gel with the ethanol extract of *S. securidaca* showed the most anti-fungal activity against all three strains.

**Conclusion:**

The *S. securidaca* extract had a significant inhibitory effect on the different species of *Candida*; however, the highest inhibitory effect was found against *C. albicans*. In order to treat candidiasis, more research is required to check the efficacy of this plant in this domain.

## Introduction

Plants are good sources of useful phytochemicals with in vitro inhibitory effects against some of the microorganisms; accordingly, they are effective in the treatment of various diseases [1]. In order to prevent and treat diseases, there has been a recent growing interest in the use of medicinal herbs, especially in Iran [2]. There has been limited success in the treatment of some human diseases (i.e., immunodeficiency disorders, such as acquired immunodeficiency syndrome [AIDS] and autoimmune diseases, and uncontrolled diabetes). This state has been even become more complicated by the increase of stress and emergence of drug resistance due to the use of broad-spectrum antibiotics, increasing the incidence of infections [3]. Regarding this, the use of natural and herbal substances and elements is of great significance in the treatment, prevention, or deceleration of the course of the disease, as well as the improvement and enhancement of the host defense system [4].

The medicinal plants are major sources of bioactive ingredients, including flavonoids, phenolic compounds, tannins, and alkaloids. Therefore, they are of particular importance in the health status of individuals and community and are widely used to treat many diseases [5]. *Securigera securidaca* L., belonging to Leguminosae family, is a herbaceous annual plant native to Western Asia, Europe, Australia, and Iran, especially in Tehran and Khuzestan provinces. This plant is also called adasolmolk in Persian [6, 7]. Various extracts derived from *S. securidaca* seeds have different therapeutic properties, such as anti-epileptic, anticonvulsant, and blood lipid-lowering. Known compounds of this *S. securidaca* include flavonoids, coumarins, sterols, saponins, and cardenolides [8, 9].

Candidiasis is a spectrum of opportunistic diseases that develop dermatological, oral, and systemic diseases, as well as various infections, especially in people with autoimmune diseases [10, 11]. These diseases vary from simple, superficial mucosal infections to dangerous systemic and even lethal disseminated infections. The etiologic agents of these diseases are the yeasts, belonging to the genus *Candida*.

Although *C. albicans* is the most common *Candida* agent responsible for infection in different clinical forms of candidiasis, other *Candida* species, including *C. tropicalis*, *C. glabrata*, *C. krusei*, *C. parapsilosis,* and *C. guilliermondii*, are also more or less isolated from patients [12]. The importance of non-*albicans* *Candida* species has increased in recent years, due to a relative resistance of several species, such as *C. tropicalis* and *C. glabrata*, to some antifungal drugs, such as fluconazole and amphotericin B [13, 14].

 On the other hand, the resistance of pathogens, prolonged course of treatment, high cost of some drugs, the incidence of complications, and occurrence of unwanted reactions have encouraged researchers to study and discover new drugs or effective alternatives, especially those from medicinal herbs [15]. Since there has been no study on the antifungal effects of *S. securidaca* on *Candida* species, the present study aimed to evaluate the effects of different extracts of this plant on three *Candida* species.

## Materials and Methods

***Materials***

The reagents and chemicals used in this study were obtained from Sigma-Aldrich (Taufirchen, Germany). These materials included ketoconazole, resazurin, Sabouraud dextrose broth, Sabouraud dextrose agar (SDA), carboxymethyl cellulose (CMC), sodium benzoate, and glycerin.

***Plant collection***

The *S. securidaca* was collected from southwestern Iran (Khuzestan province) and identified in the Khuzestan Agricultural and Natural Resources Research Center, Ahvaz, Iran (herbarium No. A151,640,100AP and ethics No. IR.AJUMS.REC.1397.742). The aerial parts of these samples were placed in a room, and then powdered, using an electric blender (Busch). 

To prepare 20 g *S. securidaca* extract, the powder was subjected to 120 ml of 80% ethanol, methanol, and butanol for 24 h, using the Soxhlet method and then filtered with the Whatman™ Qualitative Filter Paper: Grade 1 Circles. After the extract was taken, it was stored in sterilized aeration bottles at 4°C. In order to prepare dried extracts, the solution was placed at 40°C for 24 h before use [16]. 

***Preparation of conventional gel***

In the present study, *S. securidaca* gel (0.5% w/w) was prepared by CMC polymer. The CMC gel was prepared by dispersing 2.0% w/v of the polymer in water, stirred by magnetic stirring until obtaining a homogenous gel base. Subsequently, it was left for 24 h in order to allow complete swelling of CMC. The hydroalcoholic extracts (0.5 g) were dissolved in 5 ml of water and added to a portion-wise of the formed gel base to produce (0.5% w/w) drug concentration.

***Measurement of gel viscosity and pH***

Rheological experiments were carried out to examine the viscous and elastic properties of different formulations. In this investigation, Brookfield digital viscometer (Model DV-IIþ, Brookfield Engineering Laboratories, INC, Stoughton) was used at room temperature. The pH of gel formulations was measured, using a digital calibrated pH meter, in which suitable buffer solutions were utilized (Mettler Toledo's pH meter, Mumbai, India). Moreover, the appearance of gel formulations was evaluated on the basis of visual evaluation.

***Preparation of organisms***

The standard suspensions of *C. albicans* (ATCC 3153), *C. parapsilosis* (ATCC 2195), and *C. krusei* (ATCC 573) were purchased from the Mycology Research Center, Faculty of Veterinary Medicine, University of Tehran, Iran. In addition, the SDA medium was used to cultivate the strains. 

***Anti-Candida activity***

***Well diffusion assay***

The agar well diffusion procedure is widely used to investigate the antimicrobial activity of plant extracts. In order to determine the effective concentration, the inhibition zones of alcoholic extract and **vaginal gel of** *S. securidaca* against the three mentioned *Candida* species were evaluated using this technique. A well with a diameter of 6-8 mm was punched by a sterile tip in each plate; afterward, the whole surface of the medium was cultured with *Candida *species. Subsequently, the extract and gel were added to the well and incubated at 27^º^C for 24 h [17]. In this procedure, ketoconazole was used as a positive control. The inhibition zone diameter was measured, and its corresponding effective concentration was used for subsequent experiments [18].

***Determination of minimal inhibitory concentration and*** ***minimum fungicidal concentration ***

The minimal inhibitory concentration (MIC) was determined by the microdilution method [19]. Two-fold serial dilution of each extract was prepared in Sabouraud broth, and 10 µL of yeast suspension (approximately 1.5*10^8^ CFU/mL based on a standard 0.5 McFarland ) was added. The microplates were incubated at 30^º^C, and after 48 h, 50 µL of resazurin solution (0.01%) was added to each well. The plates were re-incubated for 2 h at 30^º^C, and any color changing from purple to pink was recorded as microbial growth. The lowest concentration, at which no color change occurred, was taken as the MIC. Afterward, cultures were seeded in SDA plates, incubated for 48 h at 30^º^C, and consequently minimum fungicidal concentration (MFC) was determined [20].

***Statistical analysis***

All statistical analyses were performed, using SPSS software (version 16). The inhibition zone diameter, induced by test substances, was expressed as mean±SD, and the groups were compared by one-way ANOVA and Waller-Duncan post-hoc tests.

## Results

***Anti-Candida activity***

In this study, the inhibitory effect of *S. securidaca* extracts was evaluated against *Candida* strains (Table 1). In this regard*,* the extracts showed anti-*C. albicans* activity against all tested strains. The MIC values had ranges of 156-625 and 625-1,250 µg/mL for butanol and ethanol/methanol extracts, respectively. The ethanol and methanol extracts exhibited the best antifungal potential with no significant difference between their mean MFC values (*P>0.05*) (Table 1). 

Additionally, the MFC/MIC ratios ranged from 2 to 4 for the ethanol and methanol extracts; therefore, they were fungicidal agents against all tested strains. Both fungicidal and fungistatic effects were observed for the butanol extract with an MFC/MIC range of 2-8. Since the ethanol and methanol extracts showed the strongest activity, they were chosen for further biological activity assays.

***Gel preparation***

In this study, different gels were used to evaluate the performance of various extracts of *S. securidaca *(Table 2-4).

**Table 1:** Anti-*Candida* activity of *Securigera* *securidaca* extracts

***Candida*** ** strains**	**Ethanol** ** extract** **(µ** **g** **/ml)**			**Butanol** ** extract** **(µ** **g** **/ml)**			**Methanol** ** extract** **(µ** **g** **/ml)**		
	**MIC**	**MFC**	**MFC/MIC ratio**	**MIC**	**MFC**	**MFC/MIC ratio**	**MIC**	**MFC**	**MFC/MIC ratio**
*C. parapsilosis*	625	1,250	2	156	1,250	8	625	2,500	4
*C. albicans*	1,250	2,500	2	625	2,500	4	1,250	2,500	2
*C. * *krusei *	625	2,500	4	625	1,250	2	625	1,250	2

**Table 2:** Gel formula containing *Securigera* *securidaca* extracts

**No**	**Ingredients**	**Percentage**
1	Carboxymethyl cellulose	6
2	Sodium benzoate	0.5
3	Glycerin	10
4	Extracts	4
5	Water	Quantum sufficit

**Table 3:** Physicochemical properties of vaginal gels

**Formulation**	**Texture**	**Color**	**pH value**
Control	Smooth	Colorless	8.2
Gel with ethanol extract	Smooth	Brown	6.5
Gel with butanol extract	Smooth	Brown	6.7
Gel with methanol extract	Smooth	Brown	6.3

**Table 4:** Mean inhibition zone diameter of different gel extract formulations against *Candida *species

**Formulations**	**Mean±** **SEM ** **(mm)**		
	***C. parapsilosis***	***C. albicans***	***C.*** *** krusei***
Control	00.00	00.00	00.00
Gel with ethanol extract	23±0.98	26±0.07	23.33±0.67
Gel with butanol extract	6.4±0.05	4.5±0.16	00.00
Gel with methanol extract	22.00±1.00	25±0.8	21.00±1.00
Ketoconazole	24.00±0.60	27.00±0.81	23.00±0.02

SEM: Standard error of the mean

## Discussion

In recent decades, opportunistic yeasts infections have become more important [21, 22]. It is probably due to the growing incidence and prevalence of these infections in the community, as well as among hospital-acquired infections. Debilitating diseases, such as AIDS, diabetes mellitus, and malignancies, as well as the increased use of intravenous therapy catheters, organ transplantation, anticancer drugs, broad-spectrum antibiotics, and corticosteroids, are among the underlying causes of yeast infections [23]. 

Various species have different degrees of sensitivity to antifungal drugs; for example, the sensitivity of *C. tropicalis* and *C. glabrata* to fluconazole is 4-32 times lower than that of *C. albicans *[24]. Moreover, *C. lusitaniae* has an inherent relative resistance to amphotericin B [23, 25]. In this respect, *C. albicans* is the most common cause of acute candidiasis [23, 25]. Triazoles were initially very effective against fungal infections; however, the current reports are suggestive of increased resistance against these agents. This highlights the need for extensive research, in order to assess the antifungal compound effects of different sources, especially plants [26]. The present study involved the investigation of the antifungal effects of the hydroalcoholic extract and **vaginal gel** of *S. securidaca* on different *Candida *species using well diffusion methods. In this research, the results showed that the ethanol extract had the greatest effect on *Candida* strains. 

Phytochemical research has indicated the presence of flavonoids, alkaloids, saponins, tannins, cardiac glycosides, coumarins, and 19 amino acids in *S. securidaca *[27, 28]. The analysis of high-performance liquid chromatography coupled with diode array detection, electrospray ionization, and mass spectrometry announced that the ethanolic extract contained phenolic compounds, such as various classes of phenolic acid and flavonoids [29]. 

These flavonols have anti-tumor, antioxidant, anti-allergic, analgesic, antiviral, and blood lipid-/sugar-lowering activities [29-31]. Potent cytotoxicity of some *S. securidaca* flavonoids has been demonstrated using 3-(4,5-dimethylthiazol-2-yl)-2, 5-diphenyltetrazolium bromide assay on three cancer cell lines [32]. The flavonoids act as antimicrobials in a variety of ways, such as direct antibacterial activity, synergism with antibiotics, and viral suppression [33].

For example, many researchers evaluated the antibacterial activity of flavonoids, including antibacterial activity of kaempferol and quercetin against *Propionibacterium acnes *[34], apigenin inhibitory effects against *Salmonella typhi*, *Proteus mirabilis,* and *Pseudomonas aeruginosa* [35], and selective toxicity against *Staphylococcus aureus,* including methicillin-resistant *S. aureus* and methicillin-sensitive *S. aureus* by Apigenin and Luteolin, respectively [36, 37].

In addition, there are multiple reports regarding the antimicrobial effects of some cardenolides [38], which can explain the antibacterial activity of *S. securidaca*. According to the results of this study, *S. securidaca* hydroalcoholic extract and **vaginal gel** had a significant inhibitory effect on the growth of different *Candida* strains. To a large extent, *S. securidaca* seems to be an appropriate source of antifungal compounds and can be used to treat infectious diseases.

## Conclusion

According to the findings obtained from the present study, the ethanol extract and **vaginal gel with** the ethanol extract of *S. securidaca* showed good antifungal effects, which can be attributed to the presence of phytochemicals. One of the limitations of this study was the lack of miconazole and clotrimazole usage due to poor access to both drugs. Therefore, more research is required to determine the mechanism and effect of the compounds in these extracts on fungal agents, as well as different diseases. Moreover, it is recommended to examine different extracts of *S. securidaca* by performing in vivo tests on animal models and also cell cultures. It could be useful to study the effect of these plant extracts on other fungi and bacteria and compared it with other fungal drugs.
